# Polypharmacy Is Associated With Amiodarone-Induced Hypothyroidism

**DOI:** 10.7150/ijms.61412

**Published:** 2021-08-27

**Authors:** Satoshi Yokoyama, Yuki Tanaka, Kouichi Hosomi, Mitsutaka Takada

**Affiliations:** Division of Drug Informatics, School of Pharmacy, Kindai University, 3-4-1 Kowakae, Higashiosaka City, Osaka 577-8502, Japan

**Keywords:** polypharmacy, amiodarone, hypothyroidism, data-mining, sequence symmetry analysis

## Abstract

**Background:** Amiodarone is rich in iodine, so in clinical practice amiodarone-induced hypothyroidism (AIH) is a major side effect. This drug is used in patients with arrhythmias, especially atrial fibrillation, the most common sustained arrhythmia. Polypharmacy, which can result in complex drug-drug interactions, occurs in more than 70% of the patients with atrial fibrillation. Therefore, polypharmacy may be involved in the expression of AIH. In this study, we investigated the association between polypharmacy and AIH.

**Methods:** We conducted a retrospective study using data from January 2006 to May 2020 collected from a large, organized database of prescriptions constructed by the Japan Medical Information Research Institute, Inc. (Tokyo, Japan). To investigate the association between number of prescribed drugs with amiodarone and AIH, we divided patients into two groups: polypharmacy (≥ 5 prescribed drugs) and non-polypharmacy (< 5 prescribed drugs). We then performed a sequence symmetry analysis on the two groups: incident thyroxine after incident amiodarone and incident thyroxine before incident amiodarone. Finally, we conducted a case-control study on two further groups: those prescribed thyroxine after incident amiodarone (AIH group; n=555) and those not prescribed thyroxine after incident amiodarone (non-AIH group; n=6,192).

**Results:** Sequence symmetry analysis revealed a significant association between amiodarone and thyroxine in both the polypharmacy and non-polypharmacy groups. The ranges for the adjusted sequence ratio in the two groups were 12.0-16.7 and 7.3-9.0, respectively. The case-control study showed that ≥5 prescribed drugs at the first prescription of amiodarone were found to significantly increase the odds of AIH (odds ratio: 1.48, 95% confidence interval: 1.18-1.84).

**Conclusion:** Polypharmacy was suggested as an independent risk factor for AIH. Careful assessment of the appropriateness of prescription is warranted.

## Introduction

The thyroid gland secretes thyroid hormones that play multifaceted roles in organ development and homeostatic control of fundamental physiological mechanisms 1, 2. Therefore, the thyroid gland is a vital organ. However, it is occasionally damaged by drugs such as amiodarone. Amiodarone is very rich in iodine and sometimes induces hypothyroidism 3. Severe amiodarone-induced hypothyroidism (AIH) may promote atrial fibrillation and ventricular arrhythmias, which mandate the periodic monitoring of patients through thyroid function tests 4. Therefore, it is important for clinicians to understand the risk factors of AIH.

The use of multiple medicines is called “polypharmacy” and is common among older adults. It is defined not only as the burden of taking multiple drugs, but also as the use of more medications than necessary 5. Although there is no consensus regarding the number of prescribed drugs considered to be polypharmacy, the most commonly used definition for polypharmacy was 5 or more medications daily 6. In a Japanese nationwide study, more than two-thirds of the total cases of polypharmacy were observed in individuals aged 65 and older 7. The process of deprescribing is considered to be an effective strategy to reduce the number of inappropriate drugs and consequently the number of adverse events 8,9.

Amiodarone is used for treating ventricular and atrial arrhythmia 10. The most common sustained arrhythmia is atrial fibrillation, the prevalence of which increases with age. In two large-scale clinical trials on oral anticoagulation therapy in patients with atrial fibrillation, more than 70% were using 5 or more drugs 11. Furthermore, these two trials revealed that patients with a higher number of concomitant drugs had higher rates of adverse events, such as mortality and major bleeding events. One possible cause of adverse events is the potential drug-drug interactions caused by polypharmacy. A nationwide population-based cohort study in Taiwan showed that the concurrent use of amiodarone, which is a P-glycoprotein competitor, with oral anticoagulants was associated with an increased risk of major bleeding 12. It is assumed that polypharmacy induces complex drug interactions and changes the pharmacokinetics of individual drugs. Therefore, we hypothesized that polypharmacy might be a risk factor for AIH.

Recently, with the development of information technology, big data called real-world data has been used in various studies. Because it is unethical to conduct intervention studies on the association between polypharmacy and adverse events, observational studies using real-world data are very suitable for investigating this association. In the medical field, mainly claims databases or prescription databases are available. No study on AIH with polypharmacy has been conducted using real-world data. Therefore, in this study, we investigated the impact of polypharmacy on AIH using a prescription database.

## Methods

### Study design

We conducted a retrospective study using a prescription database. First, we confirmed the association between amiodarone and AIH through data mining. Next, after dividing the enrolled patients into two groups, namely, the polypharmacy and non-polypharmacy groups, their data were mined from the prescription database to investigate the association mentioned earlier. Finally, a retrospective study was conducted to clarify the risk factors of AIH in relation to the number of prescribed drugs with amiodarone. This study was approved by the Ethics Committees of the Kindai University School of Pharmacy on April 14, 2018.

### Data source

We used a large, organized database of prescriptions constructed by a database vendor (Japan Medical Information Research Institute (JMIRI), Inc., Tokyo, Japan). This database consisted of prescriptions collected from approximately 1,500 pharmacies in Japan, excluding prescriptions from hospitals. The database contained approximately 970 million prescriptions from January 2006 to May 2020. The cumulative total number of patients in the last year was 6.2 million. The JMIRI prescription database included an encrypted personal identifier, age and gender of the patients, drug name, unique drug code and generic name, and prescribing date. All drugs were coded according to the Anatomical Therapeutic Chemical (ATC) classification. Disease name and laboratory data were not included.

### Sequence symmetry analysis

To confirm the association between the number of prescribed drugs with amiodarone and AIH, we performed sequence symmetry analysis (SSA). SSA is a method for generating hypotheses that examines only the order of exposure and outcome. SSA has high sensitivity, specificity, and positive/negative predictive value 13; therefore, it is commonly used as a pharmacovigilance tool to investigate the association between an exposure and an outcome by investigating their sequence 14. Briefly, SSA is based on a situation when an index drug is suspected of causing an outcome. SSA evaluates asymmetry in the distribution of an exposure (index drug) before and after an outcome (an adverse event that is treated by an outcome drug). Asymmetry may indicate an association between an exposure and an outcome and is evaluated by the sequence ratio (SR). The crude SR is defined as the ratio of the number of newly treated patients with an outcome drug after initiation of an index drug relative to the number before initiation. In addition, the SRs were adjusted for temporal prescribing trends in index and outcome drugs. The probability that index drugs were prescribed first, in the absence of any causal relationship, can be estimated by a so-called null-effect SR. The null-effect SR generated by the proposed model may be interpreted as a reference value for the SR. Therefore, the null-effect SR is the expected SR in the absence of any causal association after accounting for incidence trends. By dividing the crude SR by the null-effect SR, an adjusted SR corrected for temporal trends is obtained.

In this study, we selected amiodarone (ATC code: C1B) as the index drug and thyroid preparations (ATC code: H3A) as outcome drugs indicating the occurrence of AIH. All incident users of amiodarone and thyroid preparations from January 2006 to May 2020 were identified. Incidence was defined as the first prescription of amiodarone. To exclude prevalent users of amiodarone and thyroid preparations, the analysis was restricted to users whose first prescription was administered, after a run-in period of 6 months, in July 2006 or later. Waiting time distribution 15 was analyzed to ensure that SSA was restricted to incident users of amiodarone and thyroid preparations. Therefore, an identical run-in period was also applied to all patients enrolled in the cohort after June 2006. Those patients who had initiated a new treatment with amiodarone and thyroid preparations within 12-, 24-, and 36-month periods (intervals) were identified. Patients who had received their first amiodarone and thyroid preparations within the same day were not included in the determination of the SR. Next, based on the number of prescribed drugs on the index date (the day of the first prescription of amiodarone), patients were divided into two groups: the polypharmacy group (5 or more drugs) and the non-polypharmacy group (4 or less drugs), and SSA was performed in each group. The 95% confidence interval (CI) for the adjusted SR was calculated using a method for calculating exact CIs in binomial distributions 16. A significant signal was defined as the lower limit of the 95% CI being more than 1 for the adjusted SR.

### Retrospective case-control study

The JMIRI prescription database (2006/01-2020/01) contained the data of 7,082 patients who were newly administered amiodarone. The patients who were prescribed thyroxine (ATC code: H3A) before the first prescription of amiodarone (n=266) and who received thyroxine and amiodarone for the first time on the same day (n=69) were excluded. Next, we divided patients (n=6,747) into two groups: those who were prescribed thyroxine after the first prescription of amiodarone (AIH group; n=555) and those not prescribed thyroxine (non-AIH group, n=6,192). We defined the day of the first prescription of thyroxine in the AIH group and the day of the last prescription of amiodarone in the non-AIH group as the index date. We investigated the number of prescribed drugs at the first prescription of amiodarone. Furthermore, the age, sex, and drugs prescribed within three months of the index date as concomitant drugs were also investigated.

Categorical variables are presented as frequencies and proportions, while quantitative variables are presented as the median (interquartile range). Categorical and quantitative variables were compared using the Chi-square test and Mann-Whitney test, respectively. Multi-variable analysis was conducted using a multiple stepwise logistic regression model to evaluate the association between the number of prescribed drugs and AIH. All statistical tests were two-sided, with the significance set at 0.05. Statistical analyses were conducted using JMP Pro 14.2 (SAS Institute Inc., Cary, NC, USA).

## Results

### Sequence symmetry analysis using the JMIRI prescription database

The characteristics of the amiodarone users in the JMIRI prescription database are summarized in Table 1. There were three thyroid agents in the JMIRI prescription database, namely, levothyroxine sodium hydrate, liothyronine sodium, and dried thyroid. Levothyroxine sodium hydrate was mainly prescribed. The number of patients who were newly prescribed amiodarone, levothyroxine sodium hydrate, liothyronine sodium, and dried thyroid were 7,082, 47,483, 585, and 110, respectively. The majority of the patients prescribed amiodarone and levothyroxine sodium hydrate were male (68.7%) with a median age of 72 years and female (69.9%) with a median age of 67 years, respectively.

Table 2 shows the results of the SSA. The number of patients included in the SSA was 804. Adjusted SRs at all intervals were over 10.0. A significant association was observed between amiodarone and thyroxine. Table 3 shows the results of SSA in the polypharmacy and non-polypharmacy groups. Significant association was also observed at all intervals in both groups. The ranges of adjusted SRs in polypharmacy and non-polypharmacy groups were 12.0-16.7 and 7.3-9.0, respectively.

### Retrospective case-control study

The baseline characteristics of the 6,747 enrolled patients are summarized in Table 4. The numbers of patients aged 65 or more in the AIH and non-AIH groups were 453 (81.6%) and 4,723 (76.3%), respectively. The number of patients prescribed 5 or more drugs at the first prescription of amiodarone in the AIH group and that in the non-AIH group were 447 (80.5%) and 4,423 (71.4%), respectively. In both groups, more than 40% of patients were prescribed proton pump inhibitors, calcium channel blockers/agents acting on the renin-angiotensin system, or lipid-regulating/anti-atheroma preparations in combination. Table S1 shows the top 50 drugs with the highest concomitant rates. There are many drugs with the ATC codes A2B (antiulcerants), B1F (direct factor Xa inhibitors), C8A (calcium antagonists, plain), and C9C (angiotensin-II antagonists, plain).

In a multiple stepwise logistic regression model, the following factors were found to significantly increase the odds of AIH: the number of prescribed drugs at the first prescription of amiodarone ≥5 (odds ratio: 1.48, [95% CI: 1.18-1.84]), age ≥65 years (1.30 [1.04-1.63]), female (1.21 [1.00-1.63]), and concomitant drugs including cholagogues and hepatic protectors (1.74 [1.18-2.57]), anti-anemic preparations (1.43 [1.08-1.91]), and anti-gout preparations (1.47 [1.22-1.78]) (Table 5). Next, all the patients were grouped into two groups, namely, male (n=4,666) and female (n=2,081), and the same analysis was performed. The characteristics of these two groups are shown in Table S2 and S3. Multivariable analysis revealed that odds ratios of the number of prescribed drugs at the first prescription of amiodarone ≥5 were 1.43 [1.09-1.88] and 1.59 [1.09-2.32] in the male and female groups, respectively (Table S4, S5).

## Discussion

Polypharmacy is common among older adults. Unfortunately, with increased use of multiple medications, there is an increased risk for negative health outcomes such as adverse drug reactions 17. However, the extent to which polypharmacy increases the risk of negative health outcomes for individual drugs is not well understood. In this study, we evaluated the odds of hypothyroidism caused by amiodarone under polypharmacy using real-world data.

In this study, we investigated the association between amiodarone and thyroxine, which was previously reported by Pratt *et al.* 18. We have confirmed that similar results can be obtained using a different database. According to a report from the United States, hypothyroidism may develop as rapidly as two weeks or as late as 39 months after initiating amiodarone therapy 19. In contrast, several studies conducted in Japan showed that AIH is more likely to occur within 2 years of starting amiodarone 20-22. Our results only show that adjusted SRs for 1 and 2 years were higher than that for 3 years. In our study, we divided the patients into two groups based on the number of concomitant medications and performed SSA. The adjusted SR value in the polypharmacy group was higher than that in the non-polypharmacy group at all intervals. Although it is not possible to compare the risk based on the magnitude of the adjusted SR value, we presumed that polypharmacy was strongly associated with onset of AIH.

The results of our retrospective study suggest that prescription of ≥5 drugs at the first prescription of amiodarone is an independent risk factor of AIH. Amiodarone is mainly metabolized by CYP3A4 to desethylamiodarone. Amiodarone is a weak inhibitor of deiodinase, which determines the maintenance of euthyroidism at a serum level, and its metabolite desethylamiodarone is a strong inhibitor 23-25. The desethylamiodarone/amiodarone ratio might be a predictive marker for AIH 26. In this study, few patients received CYP3A4 inducers or inhibitors as concomitant drugs. However, many patients received CYP3A4 substrates such as carvedilol, lansoprazole, and apixaban concomitantly (Table S1). The change in the pharmacokinetics of amiodarone and desethylamiodarone caused by complicated drug-drug interactions due to polypharmacy may be associated with the onset of AIH. No obvious reason for the onset of AIH resulting from receiving ≥5 prescribed drugs could be suggested by this study. However, the results provided by observational studies conducted in real-world settings are representative of real-word patient populations and routine clinical practice 27.

We defined drugs prescribed within 3 months of the index date as concomitant drugs and added them to the variables. As a result, age ≥65 years, female sex, and concomitantly administered cholagogues and hepatic protectors, anti-anemic preparations, and anti-gout preparations were also identified as significant independent risk factors. Advanced age was previously reported to be a risk factor of AIH 28. Furthermore, a recent systematic review revealed that older age and female sex were risk factors for AIH 29. Generally, hypothyroidism is approximately ten times more prevalent in females than in males 30. To check whether hypothyroidism is associated with amiodarone, we divided the population into two groups (male and female) and analyzed each group. Consequently, prescription of ≥5 drugs was found to be an independent risk factor for AIH, regardless of gender. Prescribed cholagogues and hepatic protectors indicated the existence of hepatic disorders. Because amiodarone is a drug metabolized in the liver, the pharmacokinetics of amiodarone might vary due to liver dysfunction. A previous report in Japanese showed that liver dysfunction was an independent risk factor for AIH 21. In this study, iron preparations were mainly considered as “Anti-anemic preparations (ATC code: B3)”. Renal anemia may be one reason why these iron preparations were prescribed 31. The population in this study is older adults, and it is no wonder that patients with impaired renal function might be included. Decreased renal function greatly influences the clearance of drugs excreted renally. Perhaps the interaction between amiodarone and renally excreted drugs is associated with AIH. Currently, xanthine oxidase inhibitors and uricosuric agents are mainly used in gout therapy 32. Xanthine oxidase inhibitors are the usual first-line antihyperuricemic drugs used to control the serum urate. Recently, the elevation of thyroid-stimulating hormone (TSH) in patients treated with xanthine oxidase inhibitors was reported 33. A higher baseline TSH level has been reported to be a risk factor for AIH 22, 28. The combination of amiodarone with xanthine oxidase inhibitors may cause hypothyroidism additively or synergistically. Thus, these risk factors revealed in this retrospective study are supported by several previous reports.

Several potential limitations need to be considered when interpreting the results obtained in the present study. This was an observational study with a retrospective design using real-world data. Because the prescription of thyroxine was considered to be the definition of hypothyroidism, not all cases of hypothyroidism could be included in the study because subclinical hypothyroidism might not be treated with thyroxine 34. Moreover, time-dependent confounding such as deterioration of medical condition and age-related decline in renal and liver function could not be considered in the SSA. Additionally, the prescription database used in this study did not contain information such as body weight, body mass index, daily and total doses of amiodarone, blood levels of amiodarone and desethylamiodarone, and T4/TSH serum levels. The severity of heart failure and arrhythmia could not be evaluated. The drugs administered in the hospital and over-the counter drugs were unknown. We could not consider these factors in this retrospective study. In the future, a large cohort study using big data with more detailed patient information will be needed to confirm the risk of polypharmacy.

## Conclusion

In conclusion, a significant association between the number of prescribed drugs ≥5 at the first prescription of amiodarone and AIH was observed in both the SSA and retrospective study, thereby indicating polypharmacy as an independent risk factor for AIH. Regular medication reviews and deprescribing are needed to prevent this potential prescribing cascade. Furthermore, it is important for clinicians to be aware of this phenomenon. Careful assessment of the appropriateness of prescriptions is warranted as reduction in the number of medications can limit the development of AIH.

## Supplementary Material

Supplementary tables.

## Figures and Tables

**Table 1:** Characteristics of the population with amiodarone and thyroxine use

	Amiodarone		Thyroxine		
			Levothyroxine sodium hydrate	Liothyronine sodium	Dried thyroid
Prescription, n	246,657		3,677,865	10,631	12,442
Users, n	17,427		182,250	1,502	585
Incident users, n	7,082		47,483	585	110
Female, n (%)	2,217 (31.3)		33,167 (69.9)	372 (63.6)	40 (63.6)
Age, median [IQR]	72 [64-79]		67 [50-78]	57 [38-72]	72 [62-81]

Incident users: number of patients who received their first prescription of amiodarone or thyroxine IQR: interquartile range.

**Table 2:** Sequence symmetry analysis: the associations between amiodarone and thyroxine

	Incident users	Cases with thyroxine	Interval	Amiodarone→Thyroxine	Thyroxine→Amiodarone	Null-effectSR	AdjustedSR	95% CI	
			(months)					Lower	Upper
Amiodarone	7,082	804	6	178	13	1.01	13.6*	7.73	25.96
			12	311	24	1.01	12.8*	8.44	20.28
			18	382	33	1.02	11.4*	7.98	16.80
			24	430	36	1.02	11.7*	8.32	16.94
			30	456	41	1.03	10.8*	7.86	15.29
			36	488	44	1.03	10.8*	7.89	15.00

SR, sequence ratio; CI, confidence interval. *: significant signal. All patients who initiated new treatment with amiodarone and thyroxine within 36-months period were identified. Incident users: Number of patients who prescribed their first prescription of amiodarone Cases with thyroxine: Number of patients newly prescriped thyroxine in incident users

**Table 3:** Sequence symmetry analysis: the associations between amiodarone and thyroxine in polypharmacy and non-polypharmacy groups

	Incident users	Cases with thyroxine	Interval (months)	Amiodarone →Thyroxine	Thyroxine →Amiodarone	Null-effect SR	Adjusted SR	95% CI	
								Lower	Upper
Polypharmacy group	5,156	666	6	136	8	1.02	16.7*	8.25	39.51
			12	240	16	1.02	14.7*	8.86	26.11
			18	303	22	1.03	13.4*	8.70	21.74
			24	340	25	1.03	13.2*	8.78	20.68
			30	361	29	1.04	12.0*	8.20	18.14
			36	389	31	1.04	12.0*	8.33	17.94
Non-polypharmacy group	1,926	138	6	42	5	0.99	8.5*	3.35	27.41
			12	71	8	0.99	9.0*	4.31	21.55
			18	79	11	0.99	7.3*	3.84	15.12
			24	90	11	0.99	8.2*	4.39	17.09
			30	95	12	1.00	7.9*	4.34	15.91
			36	99	13	1.00	7.6*	4.26	14.81

SR, sequence ratio; CI, confidence interval. *: significant signal. All patients who initiated new treatment with amiodarone and thyroxine within 36-months period were identified. Incident users: Number of patients who prescribed their first prescription of amiodarone Cases with thyroxine: Number of patients newly prescribed thyroxine in incident users

**Table 4:** Baseline characteristics

Variables	AIH group		Non-AIH group		*p*-value
	(n=555)		(n=6,192)		
	n	(%)	n	(%)	
Age, median [interquartile range]	75	[67-81]	73	[65-80]	
Age ≥65	453	(81.6)	4,723	(76.3)	<0.01
Female	188	(33.9)	1,893	(30.6)	0.11
Prescribed drugs, median [interquartile range]	8	[5-11]	7	[4-10]	
Prescribed drugs ≥5	447	(80.5)	4,423	(71.4)	<0.01
Concomitant drugs					
Proton pump inhibitors (A2B2)	316	(56.9)	3,234	(52.2)	0.03
Cholagogues and hepatic protectors (A5)	32	(5.8)	195	(3.2)	<0.01
Inflammatory bowel disorder products (A7E)	2	(0.4)	11	(0.2)	0.29
Drugs used in diabetes (A10)	125	(22.5)	1,274	(20.6)	0.29
Anti-anemic preparations (B3)	61	(11.0)	448	(7.2)	<0.01
Calcium channel blockers/agents acting on the renin-angiotensin system (C8/C9)	348	(62.7)	3,914	(63.2)	0.81
Lipid-regulating/anti-atheroma preparations(C10)	267	(48.1)	2,656	(42.9)	0.02
Specific anti-rheumatic agents (M1C)	1	(0.2)	38	(0.6)	0.37
Anti-gout preparations (M4A)	196	(35.3)	1,614	(26.1)	<0.01
Anti-epileptics (N3A)	30	(5.4)	276	(4.5)	0.30
Anti-Parkinson drugs (N4A)	6	(1.1)	74	(1.2)	0.81
Antipsychotics (N5A)	10	(1.8)	121	(2.0)	0.80
Anti-depressants and mood stabilisers (N6A)	19	(3.4)	200	(3.2)	0.81
Anti-asthma and COPD products (R3)	41	(7.4)	411	(6.6)	0.50
Systemic antihistamines (R6)	45	(8.1)	437	(7.1)	0.36

AIH: amiodarone-induced hypothyroidism, COPD: chronic obstructive pulmonary disease, ATC: Anatomical Therapeutic Chemical Prescribed drugs: The number of prescribed drugs at the first prescription of amiodarone Concomitant drugs: Drugs prescribed within 3 months of the date that thyroxine was first prescribed in AIH group or that the last amiodarone was prescribed in non-AIH group. Words in parentheses of variables indicate the Anatomical Therapeutic Chemical code.

**Table 5:** Multivariable logistic regression analysis

Variables	Odds ratio	[95% CI]	*p*-value
Prescribed drugs ≥5	1.48	[1.18-1.84]	<0.01
Age ≥65	1.30	[1.04-1.63]	0.02
Female	1.21	[1.00-1.63]	0.04
Concomitant drugs			
Cholagogues and hepatic protectors (A5)	1.74	[1.18-2.57]	<0.01
Anti-anemic preparations (B3)	1.43	[1.08-1.91]	0.01
Specific anti-rheumatic agents (M1C)	0.24	[0.03-1.78]	0.16
Anti-gout preparations (M4A)	1.47	[1.22-1.78]	<0.01

Prescribed drugs: The number of prescribed drugs at the first prescription of amiodarone CI: confidence interval Words in parentheses of variables indicate the Anatomical Therapeutic Chemical code.

## Abbreviations

AIH: amiodarone-induced hypothyroidism
JMIRI: Japan Medical Information Research Institute
ATC: Anatomical Therapeutic Chemical
SSA: sequence symmetry analysis
SR: sequence ratio
TSH: thyroid-stimulating hormone
