# Supplementary material for: Polypharmacy Is Associated With Amiodarone-Induced Hypothyroidism
Source: Int J Med Sci. 2021 Aug 27;18(15):3574–80. doi: 10.7150/ijms.61412 (PMC8436091; doi:10.7150/ijms.61412)
Supplement: Supplementary file 1 — Supplementary tables. [file ijmsv18p3574s1.pdf]

Table S1. Top 50 most commonly used drugs

Name	ATC code	n (N=6,747)
Furosemide	C3A	2,511
Bisoprolol	C7A	2,327
Carvedilol	C7A	2,130
Warfarin	B1A	2,038
Aspirin	B1C	1,962
Spironolactone	C3A	1,657
Lansoprazole	A2B	1,513
Febuxostat	M4A	1,145
Azosemide	C3A	1,090
Rosuvastatin	C1A	1,090
Magnesium Oxide	A6A	1,088
Sennoside	A6A	963
Apixaban	B1F	891
Amlodipine	C8A	882
Esomeprazole	A2B	813
Clopidogrel	B1C	797
Atorvastatin	C1A	787
Enalapril	C9A	727
Rabeprazole	A2B	702
Tolvaptan	C3A	655
Edoxaban	B1F	628
Nicorandil	C1D	609
Rivaroxaban	B1F	598
Isosorbide	C1E	595
Allopurinol	M4A	588
Brotizolam	N5B	585
Lactic acid bacteria	A7F	506
Pitavastatin	C1A	495
Torasemide	C3A	484
Eplerenone	C2A	457
Pimobendan	C1F	447
Candesartan	C9C	433
Famotidine	A2B	431
Vonoprazan	A2B	420
Rebamipide	A2B	419
Zolpidem	N5B	418
Verapamil	C8A	395
Sodium Ferrous Citrate	B3A	389
Acetaminophen	N2B	377
Nifedipine	C8A	376
Etizolam	N5C	363
Telmisartan	C9C	359
Olmesartan	C9C	357
Losartan	C9C	336
Carbocisteine	R5C	319
Loxoprofen	M1A	296
Digoxin	C1A	274
Valsartan	C9C	270
Trichlormethiazide	C3A	266
Dabigatran	B1E	265

ATC: Anatomical Therapeutic Chemical

Table S2. Baseline characteristics (Male)

Variables	AIH group (n=367) n	(%)	Non-AIH group (n=4,299) n	(%)	*p* -value
Age, median [interquartile range]					
Age ≥65	292	(79.6)	3,103	(72.2)	<0.01
Prescribed drugs, median [interquartile range]					
Prescribed drugs ≥5	296	(80.7)	3,104	(72.2)	<0.01
Concomitant drugs					
Proton pump inhibitors (A2B2)	204	(55.6)	2,243	(52.2)	0.21
Cholagogues and hepatic protectors (A5)	21	(5.7)	133	(3.1)	<0.01
Inflammatory bowel disorder products (A7E)	2	(0.5)	7	(0.2)	0.15
Drugs used in diabetes (A10)	89	(24.3)	993	(23.1)	0.62
Anti-anemic preparations (B3)	32	(8.7)	294	(6.8)	0.17
Calcium channel blockers/agents acting on the renin-angiotensin system (C8/C9)	230	(62.7)	2,753	(64.0)	0.60
Lipid-regulating/anti-atheroma preparations(C10)	178	(48.5)	1,881	(43.8)	0.08
Specific anti-rheumatic agents (M1C)	1	(0.3)	14	(0.3)	1.00
Anti-gout preparations (M4A)	149	(40.6)	1,309	(30.5)	<0.01
Anti-epileptics (N3A)	20	(5.5)	191	(4.4)	0.37
Anti-Parkinson drugs (N4A)	3	(0.8)	50	(1.2)	0.80
Antipsychotics (N5A)	7	(1.9)	77	(1.8)	0.87
Anti-depressants and mood stabilisers (N6A)	15	(4.1)	127	(3.0)	0.23
Anti-asthma and COPD products (R3)	27	(7.4)	289	(6.7)	0.64
Systemic antihistamines (R6)	28	(7.6)	321	(7.5)	0.91

AIH: amiodarone-induced hypothyroidism, COPD: chronic obstructive pulmonary disease, ATC: Anatomical Therapeutic Chemical
Prescribed drugs: The number of prescribed drugs at the first prescription of amiodarone
Concomitant drugs: Drugs prescribed within 3 months of the date that thyroxine was first prescribed in AIH group or that the last amiodarone was prescribed in non-AIH group.
Words in parentheses of variables indicate the Anatomical Therapeutic Chemical code.

Table S3. Baseline characteristics (Female)

Variables	AIH group (n=188) n	(%)	Non-AIH group (n=1,893) n	(%)	*p* -value
Age, median [interquartile range]					
Age ≥65	161	(85.6)	1,620	(85.6)	0.98
Prescribed drugs, median [interquartile range]					
Prescribed drugs ≥5	151	(80.3)	1,319	(69.7)	<0.01
Concomitant drugs					
Proton pump inhibitors (A2B2)	112	(59.6)	991	(52.4)	0.06
Cholagogues and hepatic protectors (A5)	11	(5.9)	62	(3.3)	0.07
Inflammatory bowel disorder products (A7E)	0	(0.0)	4	(0.2)	1.00
Drugs used in diabetes (A10)	36	(19.2)	281	(14.8)	0.12
Anti-anemic preparations (B3)	29	(15.4)	154	(8.1)	<0.01
Calcium channel blockers/agents acting on the renin-angiotensin system (C8/C9)	118	(62.8)	1,161	(61.3)	0.70
Lipid-regulating/anti-atheroma preparations(C10)	89	(47.3)	775	(40.9)	0.09
Specific anti-rheumatic agents (M1C)	0	(0.0)	24	(1.3)	0.16
Anti-gout preparations (M4A)	47	(25)	305	(16.1)	<0.01
Anti-epileptics (N3A)	10	(5.3)	85	(4.5)	0.60
Anti-Parkinson drugs (N4A)	3	(1.6)	24	(1.3)	0.70
Antipsychotics (N5A)	3	(1.6)	44	(2.3)	0.52
Anti-depressants and mood stabilisers (N6A)	4	(2.1)	73	(3.9)	0.23
Anti-asthma and COPD products (R3)	14	(7.5)	122	(6.4)	0.60
Systemic antihistamines (R6)	17	(9.0)	116	(6.1)	0.12

AIH: amiodarone-induced hypothyroidism, COPD: chronic obstructive pulmonary disease, ATC: Anatomical Therapeutic Chemical
Prescribed drugs: The number of prescribed drugs at the first prescription of amiodarone
Concomitant drugs: Drugs prescribed within 3 months of the date that thyroxine was first prescribed in AIH group or that the last amiodarone was prescribed in non-AIH group.
Words in parentheses of variables indicate the Anatomical Therapeutic Chemical code.

Table S4. Multivariate logistic regression analysis (Male)

Variables	Odds ratio	[95% CI]	*p* -value
Prescribed drugs ≥5	1.43	[1.09-1.88]	0.01
Age ≥65	1.47	[1.13-1.92]	<0.01
Concomitant drugs			
Cholagogues and hepatic protectors (A5)	1.72	[1.07-2.77]	0.03
Inflammatory bowel disorder products (A7E)	3.49	[0.70-17.24]	0.13
Anti-gout preparations (M4A)	1.47	[1.18-1.84]	<0.01

Prescribed drugs: The number of prescribed drugs at the first prescription of amiodarone

CI: confidence interval

Words in parentheses of variables indicate the Anatomical Therapeutic Chemical code.

Table S5. Multivariate logistic regression analysis (Female)

Variables	Odds ratio	[95% CI]	*p* -value
Prescribed drugs ≥5	1.59	[1.09-2.32]	0.02
Concomitant drugs			
Anti-anemic preparations (B3)	1.89	[1.22-2.91]	<0.01
Anti-gout preparations (M4A)	1.57	[1.09-2.24]	0.01

Prescribed drugs: The number of prescribed drugs at the first prescription of amiodarone
CI: confidence interval
Words in parentheses of variables indicate the Anatomical Therapeutic Chemical code.
